# Anterior mitral leaflet laceration using the MitraCut technique for transapical transcatheter Tendyne implantation after unsuccessful Carillon indirect annuloplasty: a case report

**DOI:** 10.1093/ehjcr/ytae035

**Published:** 2024-01-31

**Authors:** Nunzio Davide de Manna, Andreas Martens, Marieke Jüttner, Dominik Berliner, Johann Bauersachs, Arjang Ruhparwar, Tibor Kempf, Fabio Ius

**Affiliations:** Department of Cardiothoracic, Transplant and Vascular Surgery, Hannover Medical School, Carl-Neuberg Strasse 1, Hannover 30625, Germany; Department of Cardiothoracic, Transplant and Vascular Surgery, Hannover Medical School, Carl-Neuberg Strasse 1, Hannover 30625, Germany; Department of Anesthesiology and Intensive Care Medicine, Hannover Medical School, Hannover, Germany; Department of Cardiology and Angiology, Hannover Medical School, Hannover, Germany; Department of Cardiology and Angiology, Hannover Medical School, Hannover, Germany; Department of Cardiothoracic, Transplant and Vascular Surgery, Hannover Medical School, Carl-Neuberg Strasse 1, Hannover 30625, Germany; Department of Cardiology and Angiology, Hannover Medical School, Hannover, Germany; Department of Cardiothoracic, Transplant and Vascular Surgery, Hannover Medical School, Carl-Neuberg Strasse 1, Hannover 30625, Germany

**Keywords:** Tendyne, Transcatheter mitral valve implantation, Mitral valve regurgitation, Left ventricular outflow tract obstruction, Case report

## Abstract

**Background:**

The introduction of a transapical transcatheter beating heart replacement system has significantly expanded therapeutic options for patients with severely diseased mitral valves, particularly those ineligibles for traditional surgery or transcatheter repair. However, challenges, such as left ventricular outflow tract obstruction (LVOT-O) and the risk of dynamic systolic anterior motion (SAM) in cases with elongated anterior mitral leaflet (AML) post-prosthesis implantation, impede the widespread adoption of transcatheter mitral valve replacement (TMVR).

**Case summary:**

In 2022, a 75-year-old male with severe mixed-genesis mitral regurgitation (MR) underwent Carillon Mitral Contour System annuloplasty. Recurrent heart failure admissions (New York Heart Association IV) and prohibitive risk for open-heart surgery (European System for Cardiac Operative Risk Evaluation II 8.27%) prompted evaluation for Tendyne TMVR with the MitraCut technique. This beating heart transapical approach involved scissor-mediated splitting of the elongated 27 mm AML, essential for mitigating LVOT-O risk and dynamic SAM. The screening echocardiogram revealed the poorly tethered AML near the thickened septum at the simulated neo-LVOT site.

**Discussion:**

This case underscores the intricate management challenges associated with severe MR, highlighting the successful application of the MitraCut technique as a viable alternative in high-risk scenarios. The imperative for further research and clinical studies is emphasized to comprehensively elucidate outcomes and safety parameters, providing valuable insights for refining TMVR applications within this context.

**Learning points**

- To present the application of an implantation of a tether-based Tendyne transcatheter mitral valve replacement (TMVR) system using the MitraCut technique as a valuable and safe alternative for preventing left ventricular outflow tract obstruction (LVOT-O) in high-risk TMVR cases.
- To contribute to evidence-based decision-making in challenging TMVR scenarios by showcasing the potential of the MitraCut technique for mitigating systolic anterior motion (SAM) risk and dynamic LVOT-O.

## Primary specialties involved other than cardiology

Cardiology, cardiothoracic surgery, anaesthesia, intensive care.

## Introduction

Transcatheter mitral valve replacement (TMVR) has expanded the treatment landscape for patients with severely diseased mitral valves (MV) ineligible for conventional surgery.^1^ Nevertheless, the broad acceptance of TMVR has been limited by the occurrence of life-threatening left ventricular outflow tract obstruction (LVOT-O), often due to elongated and poor tethered anterior mitral valve leaflet (AML). The implant of a TMVR prosthesis fixes the AML in a diastolic position and displaces it towards the neo-LVOT tract, which can predispose patients to the development of systolic anterior motion (SAM) and dynamic LVOT-O, even when the LVOT area is considered sufficient.^2^

Effectively addressing the challenge of preventing LVOT-O requires innovative solutions, given the technical complexity, time-consuming, and substantial additional risks associated with current approaches. In this case report, we showcase the successful and safe implementation of the MitraCut technique^3,4^ as a surgical measure to prevent LVOT-O during TMVR. Notably, this technique demonstrated efficacy in a patient who had previously undergone indirect mitral annuloplasty.

## Summary figure

**Figure:**
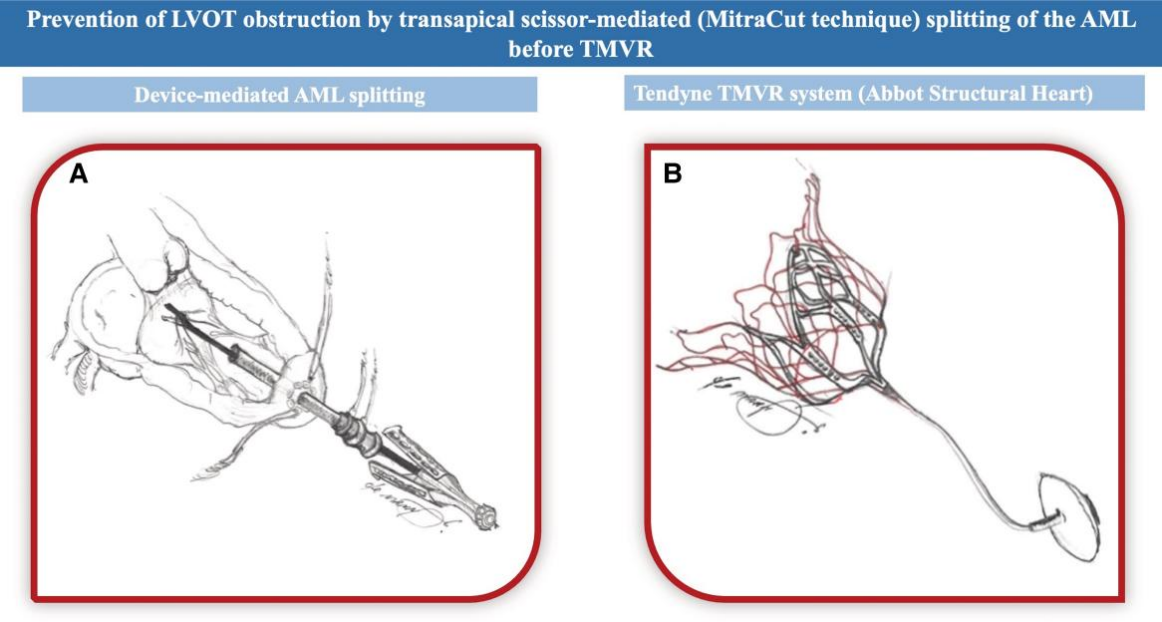
Prevention of LVOT obstruction during TMVR using scissor-mediated (MitraCut technique) splitting of the AML. (*A*) The MitraCut technique employed a 24-F Gore DrySeal Flex sheath (W.L. Gore & Associates, Inc.) that was shortened for a length of 10 cm to improve handling of the scissors and was inserted over a standard-length dilator at the transapical access site. A straight endoscopic scissor (Ceramo HCR, MRT-2; Fehling Surgical Instrument, Inc.) (length, 350 mm; shaft, 5.0 mm) is advanced through the sheath, and its tips are positioned within the A2 scallop region, guided by TEE and fluoroscopy. (*B*) The Tendyne TMVR system (Abbott Structural Heart, Santa Clara, CA, USA). AML, anterior mitral leaflet; LVOT, left ventricular outflow tract; TMVR, transcatheter mitral valve replacement; TEE, transoesophageal echocardiography.

## Case presentation

A 75-year-old male patient (*Table 1*) with a complex medical history, including atrial fibrillation, pacemaker implantation for bradycardia, severe coronary artery disease with prior stent placement, and severe mitral regurgitation (MR), had been treated with a Carillon 12/18/80 mm Mitral Contour System (Cardiac Dimensions, Kirkland, WA) in December 2022. He presented with recurrent hospitalizations due to acute heart failure [New York Heart Association (NYHA) IV].

**Table 1:** Timeline of events

July 2020	Single-chamber VVI pacemaker implantation for permanent atrial fibrillation with symptomatic bradycardias (syncope).
December 2022	The patient underwent indirect mitral annuloplasty using the Carillon 12/18/80 mm Mitral Contour System (Cardiac Dimensions, Kirkland, WA) for severe mitral regurgitation (MR) mixed-genesis (effective regurgitant orifice area, 0.53 cm^2^; regurgitant volume, 89 mL).
February 2023	The patient complained of recurrent retrosternal pain. The coronary angiography revealed a severe coronary artery disease. A drug-eluting stent was implanted for the left anterior coronary artery after intracoronary lithoplasty and a dual anti-platelet therapy was started.
May 2023	The patient was referred to our hospital for acute heart failure, New York Heart Association (NYHA) IV. Transthoracic echocardiography (TTE) showed LVEF 65% and severe MR (vena contracta, 0.8 cm; EROA, 0.36 cm^2^; R-Vol 55 mL) with pulmonary hypertension (right ventricular systolic pressure 91 mmHg).
July 2023	After multidisciplinary cardiac team discussions, the patient underwent off-label dedicated beating heart transapical tether-based standard profile (SP) 33M Tendyne TMVR system (Abbot Structural Heart) and a scissor-mediated splitting of the anterior mitral valve leaflet (AML) (MitraCut technique) due to anticipated risk for left ventricular outflow tract obstruction (LVOT-O) with a cross-sectional neo-LVOT area (399.6 mm^2^ in end-systole) and an elongated AML (27 mm).
Days 1–2	The patient was transferred to the ICU after the procedure. The post-operative TTE showed a well-functioning transcatheter heart valve without paravalvular leak and thrombus or LVOT-O.

The patient’s regular medication regimen included oral anticoagulation, angiotensin-2 receptor blockers, phosphodiesterase-5 inhibitors, diuretics, a sodium/glucose co-transporter 2 inhibitor, sodium bicarbonate, a proton pump inhibitor, and statins. Baseline functional assessment revealed pronounced peripheral oedema, reliance on a walker for ambulation, dyspnoea at rest, and left thoracic pain with minimal physical exertion. Additionally, the patient presented with chronic kidney disease [Kidney Disease: Improving Global Outcomes (KDIGO) stage IV], severe secondary pulmonary artery hypertension (right ventricle systolic pressure of 91 mmHg), and severe obesity [body mass index (BMI) 44.1 kg/m²].

Upon admission, vital signs were recorded as follows: blood pressure (106/52 mmHg), heart rate (80 b.p.m.), respiratory rate (24 breaths per minute), and temperature (36°C).

**Figure 1:**
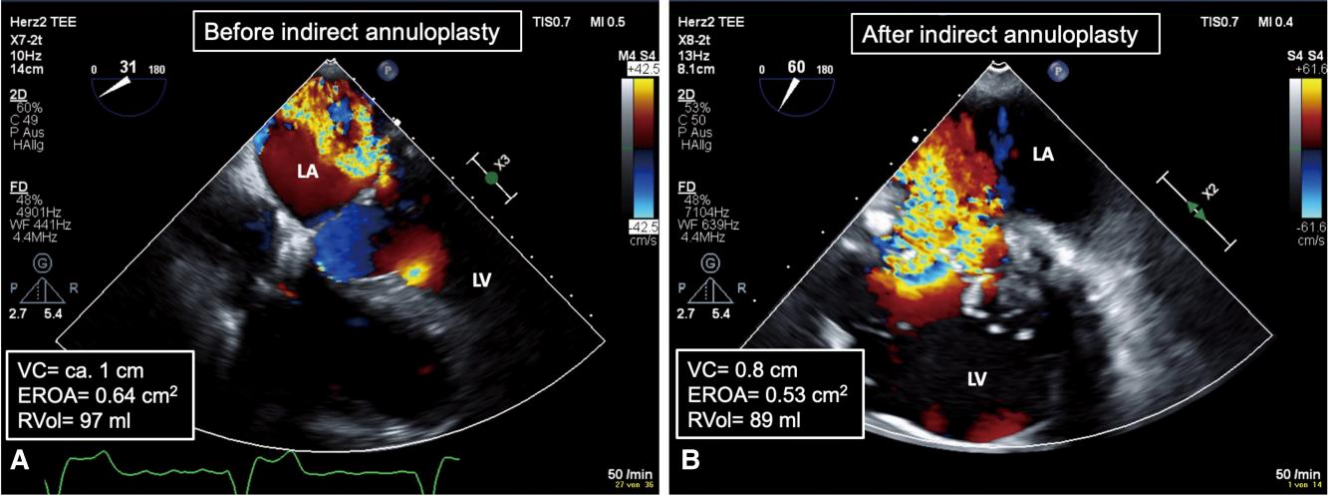
Pre-operative 2D TEE with colour Doppler images before and after Carillon device implantation. (*A*) Presence of severe MR prior to the indirect annuloplasty procedure. (*B*) Unfavourable outcome of the procedure, characterized by severe and persistent MR. 2D TEE, two-dimensional transoesophageal echocardiography; VC, vena contracta; MR, mitral valve regurgitation; EROA, effective regurgitant orifice area; R-Vol, mitral valve regurgitant volume.

Transoesophageal echocardiography (TEE) revealed a left ventricular ejection fraction of 65% and severe mixed-genesis MR (vena contracta, 0.8 cm; EROA, 0.53 cm^2^; R-Vol 89 mL), resulting from severe mitral annulus dilatation (42 mm) and marked left atrial enlargement (left atrial volume index, 93.3 mL/m^2^) (*Figure 1A* and *B*). After multidisciplinary heart team assessment, repeated treatment of the severe MR was indicated, but the patient was considered at prohibitive risk for open-heart surgery [European System for Cardiac Operative Risk Evaluation (EuroSCORE) II] 8.27%). Since transcatheter edge-to-edge MV repair was not considered feasible, due to the unfavourable anatomy of the MV leaflets (short, <5 mm, and degenerated posterior mitral leaflet), the decision was made to proceed with implanting a tether-based Tendyne TMVR system.

**Figure 2:**
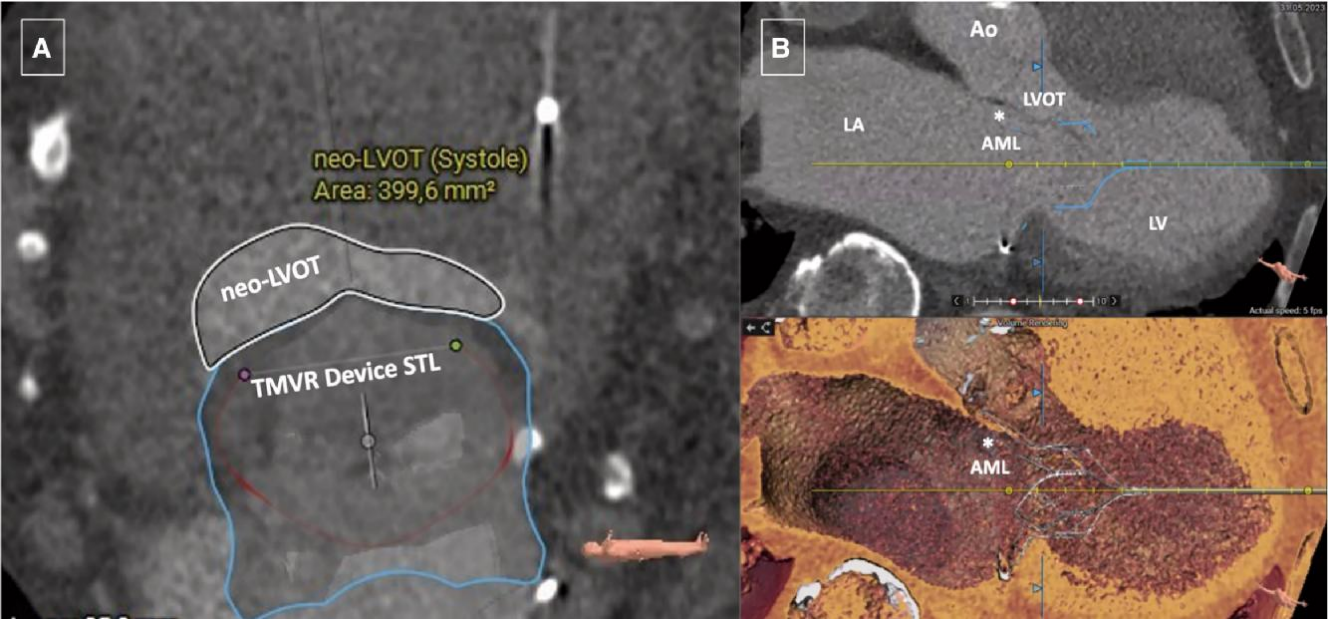
Pre-procedural cardiac CT assessment, following the deployment of a stereolithographic file of a SP 33M Tendyne system (Abbott Structural Heart, Santa Clara, CA, USA), highlights the risk of dynamic LVOT obstruction and SAM. (*A*) Predicted neo-left ventricular outflow tract (neo-LVOT) area after virtual device implantation at end-systole. (*B*) Elongated AML (asterisk). Ao, aorta; LA, left atrium; LV, left ventricle; STL, stereolithography; TMVR, transcatheter mitral valve replacement; CT, computed tomography; LVOT, left ventricle outflow tract; SP, standard profile; SAM, systolic anterior motion of the mitral valve; AML, anterior mitral valve leaflet.

**Figure 3:**
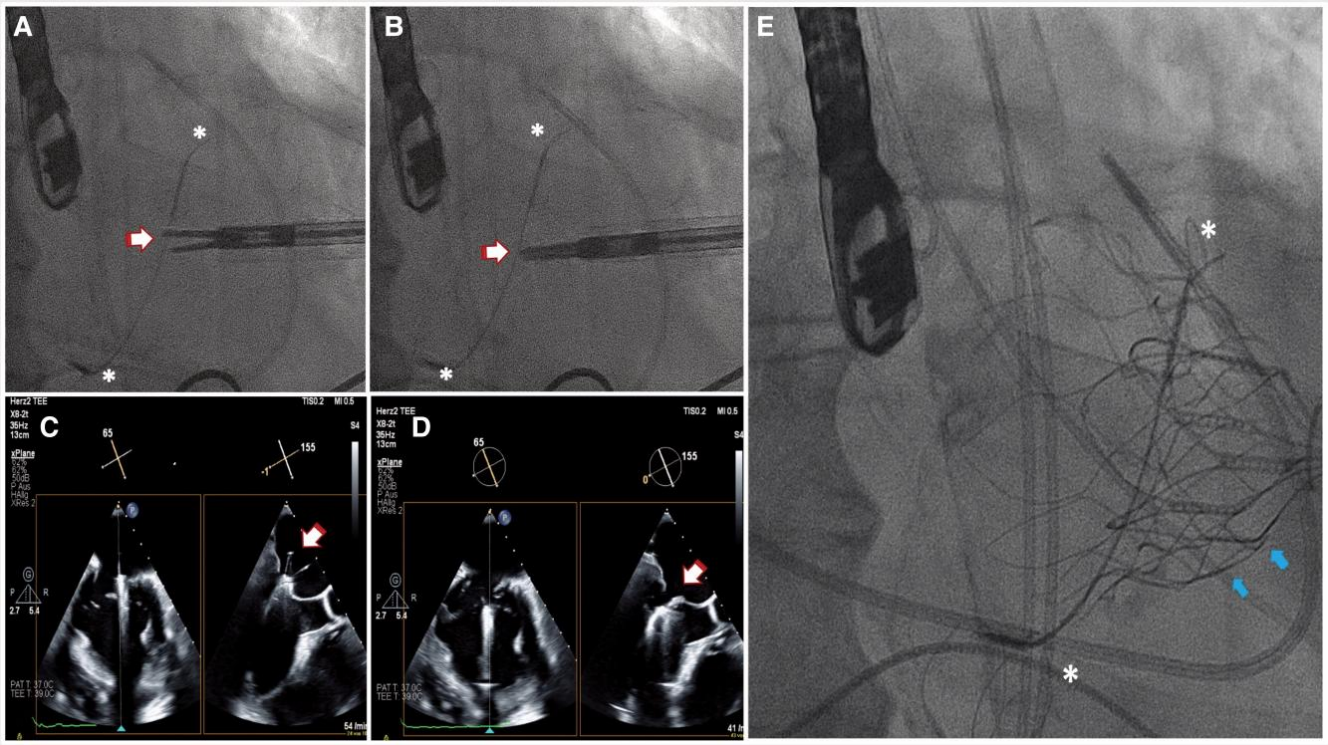
Simultaneous intraprocedural TEE and fluoroscopic procedural images. (*A–B*) Fluoroscopic images show the Carillon device (asterisks), the sheath, and the endoscopic scissor just before and after the laceration of the anterior mitral leaflet (arrows). (*C–D*) 2D TEE multi-plane imaging (X-plane) guidance shows the simultaneous bi-commissural and LVOT views before and after the MitraCut procedure with scissor blades (indicated by the arrow). (*E*) Fluoroscopic views of the Carillon® Mitral Contour System™ device implanted in the coronary sinus (asterisks) and TMVR (arrows) with a SP 33M Tendyne system (Abbott Structural Heart, Santa Clara, CA, USA). 2D TEE, two-dimensional transoesophageal echocardiography; TMVR, transcatheter mitral valve replacement; SP, standard profile; LVOT, left ventricular outflow tract.

Following standardized computer tomographic (CT) segmentation and virtual device implantation, the patient was considered eligible to receive a standard profile (SP) 33M transcatheter heart valve. However, due to the anticipated risk of LVOT-O with a cross-sectional neo-LVOT area of 399.6 mm^2^ in end-systole and an elongated, poorly supported AML (27 mm) nearly striking the septum at the simulated neo-LVOT (*Figure 2*), the decision was made to perform a dedicated beating heart transapical scissor-mediated splitting of the AML (MitraCut technique), just before TMVR (Summary figure and *Figures 3A–D*).

**Figure 4:**
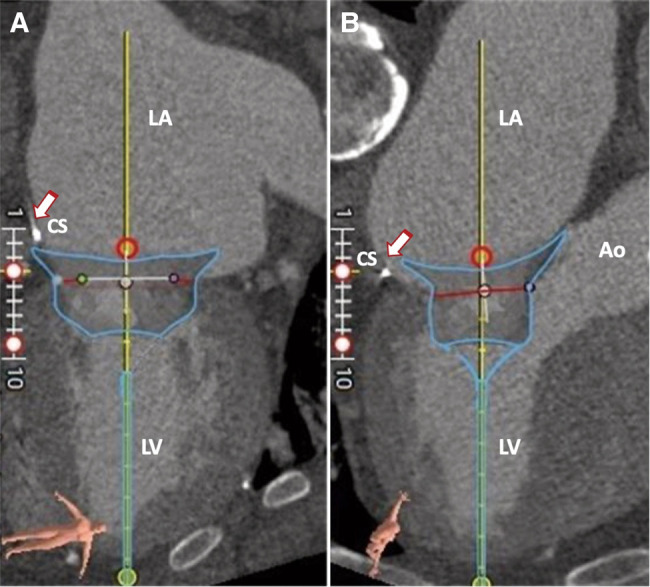
Pre-procedural cardiac CT scan and Tendyne device simulation. (*A*) Multiplanar reformation of the left-sided cardiac chambers with emphasis on the Carillon ring in the CS (arrow). (*B*) Three-chamber long axis view. Ao, aorta; LA, left atrium; LV, left ventricle; CS, coronary sinus; CT, computed tomography.

Under general anaesthesia, standard beating heart transapical access was obtained. A shortened 24-F Gore DrySeal Flex sheath (W.L. Gore & Associates, Inc.) was employed for improved handling and inserted over a standard-length dilator at the transapical access site (Summary figure A). Endoscopic scissor (Ceramo HCR, MRT-2; Fehling Surgical Instrument, Inc.) insertion was guided by three-dimensional TEE and fluoroscopic evaluation, ensuring accurate alignment of the scissor tips for the AML splitting in the A2 scallop region (*Figure 3A–D*). Subsequently, the sheath was exchanged for the Tendyne delivery system, and the prosthesis was successfully implanted (*Figure 3E*). The final TEE evaluation did not show any paravalvular leakage (PVL) or any LVOT-O. Notably, the prior indirect annuloplasty with Carillon device did not impact the deployment or seating of the Tendyne valve (*Figure 4*).

After the procedure, the patient was transferred to the intensive care unit, where he initially required inotropic support and nitric oxide (20 ppm) ventilation for concomitant pulmonary artery hypertension. The patient was extubated 25 h after the procedure and was transferred to the normal station and discharged from the hospital 14 days after the procedure, respectively, without any cardiac complication. The post-operative TTE showed a well-functioning transcatheter heart valve (mean gradient, 2 mmHg) without PVL, prosthesis thrombosis, and LVOT-O.

At the 6-month follow-up, the patient is alive and maintains stable clinical conditions.

## Discussion

This case report illustrates the complex management of severe MR in a high-risk patient with multiple comorbidities, employing innovative techniques. The successful application of the MitraCut technique highlights its potential to mitigate the risks of SAM and dynamic LVOT-O in complex TMVR scenarios.^5^

Russo *et al*.^6^ have provided comprehensive insights into LVOT-O predictors after TMVR, involving various factors such as prosthesis-, patient-, and procedure-related elements. However, they all converged around a common factor, which is the critical threshold of the neo-LVOT area that varies from 1.7 to 1.9 cm^2^.

**Figure 5:**
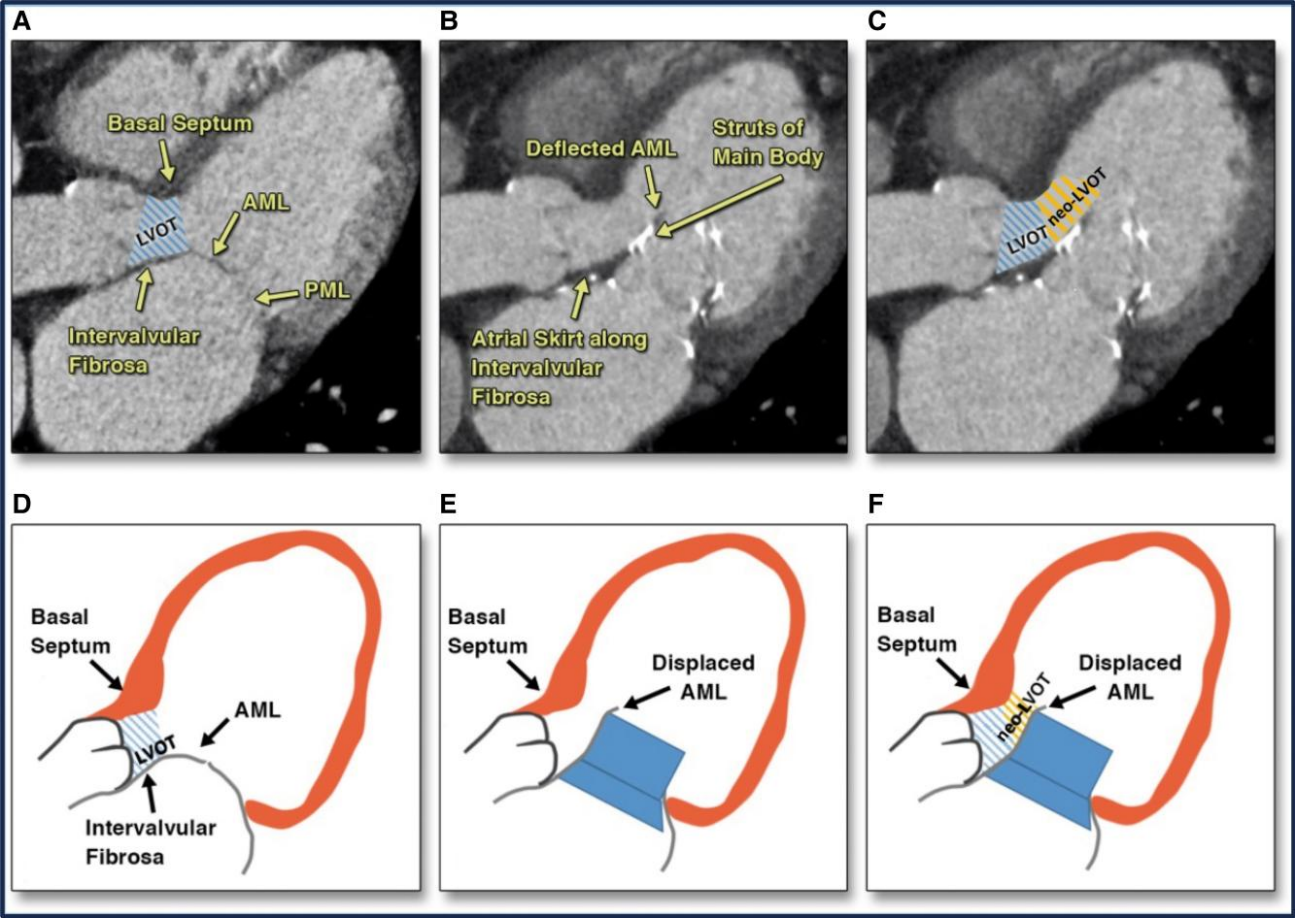
(*A–C*) CT images assessment of neo-LVOT area with a stereolithographic Tendyne device model for TMVR. (*D–F*) Corresponding graphical illustrations of AML displacement towards the neo-LVOT after a simulated Tendyne system implantation. The TMVR device pins the native AML open, displacing it towards the septum, thereby creating a neo-LVOT, confined by the displaced AML, the stent of the TMVR, and the basal-mid anteroseptal left ventricular wall (*A*, *F*). CT, computed tomography; LVOT, left ventricular outflow tract; TMVR, transcatheter mitral valve replacement; AML, anterior mitral valve leaflet; PML, posterior mitral valve leaflet.

Although scissor-mediated AML laceration has been well-documented in small LVOT area cases, there is limited scientific evidence supporting the use of the MitraCut technique before Tendyne system implantation in cases with an adequate LVOT area, especially following Carillon Mitral Contour System implantation. In our case report, despite a sufficiently predicted neo-LVOT area (almost 400 mm^2^), placing the Tendyne system without prior AML splitting in a patient with an elongated and poorly supported AML would have fixed the AML in diastole by moving it towards the LVOT, potentially resulting in SAM and dynamic LVOT-O (*Figure 5D* and *F*).

At our institution, using the pre-operative imaging, an AML value with a length ≤ 25 mm is not considered a risk of SAM. Furthermore, following the recommendations from Abbott Tendyne Vascular, patients should initially be evaluated for a SP valve. In cases where suitable anatomy is lacking, consideration of a low-profile valve is recommended. For this group of patients, the predicted neo-LVOT, averaged over end-systole and end-diastole, should be larger than 325 mm².

The current TMVR literature comprises heterogeneous reports about the applicability of the AML laceration to prevent LVOT-O using the LAMPOON technique or alcohol septal ablation for neo-LVOT modulation.^7^ However, the MitraCut technique may be superior to other wire-based interventional techniques in terms of vascular complications, extensive physician expertise, and decreased fluoroscopic time. Particularly, the direct transapical approach allows for more precise positioning of the AML cut. Instead, in the LAMPOON technique, the trajectory of the pull of the ‘flying V’ with electrocautery may result in AML laceration without ideal alignment with the LVOT.^7^ Lastly, the likelihood of apical access complications associated with the MitraCut approach is reduced due to the use of a smaller guiding sheath (26Fr) compared with the inserted valve system (34Fr).

Despite ongoing efforts for developing dedicated TMVR devices and distinct implantation techniques, along with reliable pre-procedural imaging,^8,9^ LVOT-O remains a major exclusion criterion in native TMVR.^10^ In recent clinical trials, around 50% of patients were excluded based on LVOT-O and/or anatomical considerations.^11–13^ Even among individuals with a failing bioprosthesis, such as MV-in-valve, valve-in-ring, or those with mitral annulus calcification, the risk of neo-LVOT-O exceeds that observed in native TMVR cases, with rates ranging from 2.2% to 2.6%.^14^ Thus, the MitraCut technique offers a promising solution to address this challenge and to further expand the TMVR application.

Recently, the ShortCut^TM^ mitral device (Pi-Cardia Ltd.) has been presented as promising tool for mechanical AML laceration, effectively preventing anticipated LVOT-O during TMVR.^15^ This contributes to the increasing endorsement of MitraCut and the ShortCut^TM^ (Pi-Cardia Ltd.) device in a TA approach, providing a secure and valuable alternative for patients with MV disease who have limited treatment options.

## Conclusion

The successful use of the MitraCut technique highlights its potential in high-risk scenarios, contributing to evidence-based decision-making in challenging TMVR situations. Further studies are needed to expand its utility, offering hope for improved outcomes in patients with limited treatment options.

## Data Availability

The data underlying this article are available in the article.

## Lead author biography

Dr Davide de Manna is an accomplished cardiothoracic resident currently pursuing additional training at Hannover Medical School (Hannover, Germany) to enhance his expertise in endovascular and transcatheter skills. He completed his MD degree in 2016 at University of Rome ‘Tor Vergata’ and subsequently embarked on a cardiothoracic surgery internship at the University of Verona (Italy) from 2018 to 2023. Driven by his passion for structural heart disease, transplantation, and robotic surgery, he has shown dedication to advancing his knowledge and skills in this field.

## References

[1] Alperi A, Granada JF, Bernier M, Dagenais F, Rodés-Cabau J. Current status and future prospects of transcatheter mitral valve replacement: JACC state-of-the-art review. J Am Coll Cardiol 2021;77:3058–3078. 34140110 10.1016/j.jacc.2021.04.051

[2] Charitos EI, Busch N, Renker M, Liakopoulos OJ, Fischer-Rasokat U, Colli A, et al Direct, transapical, scissors-mediated LAMPOON: keeping it simple!. JACC Cardiovasc Interv 2023;16:991–992. 37100564 10.1016/j.jcin.2022.12.014

[3] Lisko J, Kamioka N, Gleason P, Byku I, Alvarez L, Khan JM, et al Prevention and treatment of left ventricular outflow tract obstruction after transcatheter mitral valve replacement. Interv Cardiol Clin 2019;8:279–285. 31078183 10.1016/j.iccl.2019.02.005 PMC10652043

[4] Andreas M, Kerbel T, Chaplygin A, Simon P, Bartunek A, Mach M. Novel technique to prevent outflow tract obstruction in transapical transcatheter mitral valve replacement: the MitraCut technique. JTCVS Tech 2023;18:53–56. 37096076 10.1016/j.xjtc.2023.01.019 PMC10122151

[5] Mach M, Kerbel T, Poschner T, Bartunek A, Sauer J, Laufer G, et al Mitral leaflet cutting before transcatheter mitral valve replacement: the Mitra-Cut technique. JACC Cardiovasc Interv 2022;15:2107–2108. 36265945 10.1016/j.jcin.2022.08.035

[6] Russo G, Gennari M, Gavazzoni M, Pedicino D, Pozzoli A, Taramasso M, et al Transcatheter mitral valve implantation: current status and future perspectives. Circ Cardiovasc Interv 2021;14:e010628. 34407621 10.1161/CIRCINTERVENTIONS.121.010628

[7] Babaliaros VC, Greenbaum AB, Khan JM, Rogers T, Wang DD, Eng MH, et al Intentional percutaneous laceration of the anterior mitral leaflet to prevent outflow obstruction during transcatheter mitral valve replacement: first-in-human experience. JACC Cardiovasc Interv 2017;10:798–809. 28427597 10.1016/j.jcin.2017.01.035 PMC5579329

[8] Rudzinski PN, Dzielinska Z, Witkowski A, Konka M, Katarzyna KL, Demkow M. Transcatheter valve-in-valve implantation in a degenerated mitral bioprosthesis using a trans-septal anterograde approach and 3-D transesophageal echocardiography guidance. J Heart Valve Dis 2016;25:90–92. 27989091

[9] Rudzinski PN, Leipsic JA, Schoepf UJ, Dudek D, Schwarz F, Andreas M, et al CT in transcatheter-delivered treatment of valvular heart disease. Radiology 2022;304:4–17. 35638923 10.1148/radiol.210567

[10] Reid A, Ben Zekry S, Turaga M, Tarazi S, Bax JJ, Wang DD, et al Neo-LVOT and transcatheter mitral valve replacement: expert recommendations. JACC Cardiovasc Imaging 2021;14:854–866. 33248959 10.1016/j.jcmg.2020.09.027

[11] Regueiro A, Granada JF, Dagenais F, Rodés-Cabau J. Transcatheter mitral valve replacement: insights from early clinical experience and future challenges. J Am Coll Cardiol 2017;69:2175–2192. 28449780 10.1016/j.jacc.2017.02.045

[12] Bapat V, Rajagopal V, Meduri C, Farivar RS, Walton A, Duffy SJ, et al Early experience with new transcatheter mitral valve replacement. J Am Coll Cardiol 2018;71:12–21. 29102689 10.1016/j.jacc.2017.10.061

[13] Urena M, Vahanian A, Søndergaard L. Patient selection for transcatheter mitral valve implantation: why is it so hard to find patients? EuroIntervention 2018;14:AB83–AB90. 30158099 10.4244/EIJ-D-18-00510

[14] Yoon SH, Whisenant BK, Bleiziffer S, Delgado V, Dhoble A, Schofer N, et al Outcomes of transcatheter mitral valve replacement for degenerated bioprostheses, failed annuloplasty rings, and mitral annular calcification. Eur Heart J 2019;40:441–451. 30357365 10.1093/eurheartj/ehy590

[15] Ludwig S, Kalbacher D, Waldschmidt L, Schaefer A, Modine T, Dvir D, et al Prevention of LVOT obstruction by device-mediated laceration of the anterior mitral valve leaflet during TMVR. JACC Case Rep 2023;16:101873. 37396329 10.1016/j.jaccas.2023.101873 PMC10313487

